# The structure of magnesium stearate trihydrate determined from a micrometre-sized single crystal using a microfocused synchrotron X-ray beam

**DOI:** 10.1107/S2052520623005607

**Published:** 2023-07-08

**Authors:** Mikkel Herzberg, Toms Rekis, Anders Støttrup Larsen, Ana Gonzalez, Jukka Rantanen, Anders Østergaard Madsen

**Affiliations:** a Department of Pharmacy, University of Copenhagen, Universitetsparken 2, 2100 Copenhagen, Denmark; b BioMAX, MAX IV, Fotongatan 2, 224 84 Lund, Sweden; IISER Kolkata, India

**Keywords:** magnesium stearate trihydrate, molecular crystals, pharmaceutical solids

## Abstract

Crystalline magnesium stearate has been extensively used as an additive in pharmaceutical and other industries for decades. However, the lack of suitably large crystals has hindered the determination of the crystal structure and thereby a more fundamental understanding of the structure–functionality relationship. Presented here is the structure of magnesium stearate trihydrate as determined from X-ray diffraction data of a micrometre-sized single crystal measured at a fourth-generation synchrotron facility. Despite the small size of the single crystals and the weak diffraction, it was possible to determine the positions of the non-hydrogen atoms reliably. Periodic dispersion-corrected density functional theory calculations were used to obtain the positions of the hydrogen atoms playing an important role in the overall organization of the structure via a hydrogen-bond network.

## Abstract 2

The structure of the pharmaceutical lubricant magnesium stearate has been determined from micrometre-sized single crystals at a fourth-generation synchrotron.

## 1. Introduction

**Figure 1:**
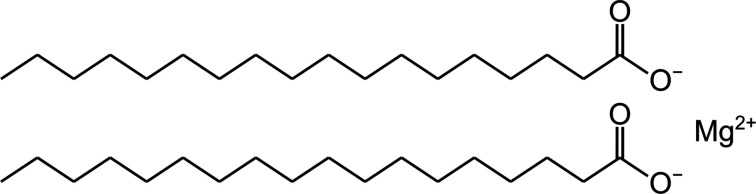
The chemical structure of magnesium stearate.

Magnesium stearate (Fig. 1 ▸) is the most commonly used additive in the manufacture of solid-state pharmaceuticals, *e.g.* tablets (Weiner & Kotkoskie, 2019 ▸; Zarmpi *et al.*, 2017 ▸). These solid products cover approximately 70% of the pharmaceutical market. As it is also found in various foods and detergents, it is one of the most widely used additives in powder-based products. Magnesium stearate is known to form mono-, di- and trihydrates, as well as an anhydrous crystalline phase (Sharpe *et al.*, 1997 ▸). Despite its wide adoption, no crystal structures are available, even though X-ray diffractograms of different magnesium stearate solid forms were described more than 70 years ago (Smith & Ross, 1946 ▸). Around the same time, the use of magnesium stearate in solid-state pharmaceuticals increased to the level that several pharmacopoeias published a compound monograph of it (US Pharmacopeial Convention, 1950 ▸; Danske Farmakopékommission, 1963 ▸).

The absence of accurate crystal structures of the magnesium stearate phases means that fundamental understanding of the compound is severely lacking, which is a barrier in the development of novel pharmaceuticals. The main reason for the missing crystal structures might be that it is problematic to obtain large enough single crystals suitable for X-ray diffraction studies due to the compound’s extremely low solubility in a variety of solvents (Palit & McBain, 1947 ▸). Attempts to elucidate the structures from powder X-ray diffraction patterns have also proven to be unsuccessful (Sharpe *et al.*, 1997 ▸; Bracconi *et al.*, 2003 ▸). In addition, pure magnesium stearate is not available commercially and therefore it has to be synthesized and purified in situ (Delaney *et al.*, 2017 ▸).

Adherence of powder mixtures onto production equipment is one of the main obstacles in the development and commercial production of pharmaceuticals. Magnesium stear­ate can help overcome these issues if used carefully with the right concentration, mixing time, hydration level and composition (Rowe *et al.*, 2009 ▸). Even small changes in these parameters can lead to disastrous problems during manufacturing (Miller & York, 1988 ▸; Okoye *et al.*, 2012 ▸) or to variation in drug release (Billany & Richards, 1982 ▸; Démuth *et al.*, 2016 ▸). This is problematic, since elevated temperatures or changes in humidity can easily lead to solid-state transformations between the hydrated forms which have varying lubrication properties (Li & Wu, 2014 ▸).

In recent years, several approaches to structure determination from micrometre- and nanometre-sized crystals have seen improvements in methodology and have thereby provided new opportunities. The use of simulated annealing techniques for structure determination from powder diffraction is one such technique (Harris *et al.*, 2001 ▸). In situations where phase purity cannot be assumed, electron diffraction, in the form of 3D electron diffraction techniques, has shown great results (Gemmi *et al.*, 2019 ▸). Lastly, micrometre- and nanofocused synchrotron radiation is available and has provided protein (Warne *et al.*, 2008 ▸) and oligopeptide structures (Nelson *et al.*, 2005 ▸) of utmost biological importance, although this approach has been used to a lesser extent for small-molecule research. Whereas the crystal sizes found in the magnesium stearate system are sufficiently small to use electron diffraction techniques, the system is sensitive to radiation damage because of the lack of aromaticity (Gruene *et al.*, 2021 ▸).

In the present work, we present the crystal structure of magnesium stearate trihydrate as determined from a microfocus synchrotron X-ray diffraction experiment on a micrometre-sized single-crystal. We show that, with the right experimental design and access to a state-of-the-art synchrotron facility, it is possible to overcome the crystal size limitation and successfully elucidate the structure.

## 2. Experimental

### 2.1. Preparation of pure crystalline magnesium stearate by salt formation

Pure magnesium stearate powder was prepared by dispersing 0.2 mol stearic acid (99%, Sigma–Aldrich) in deionized water (1200 ml, Milli-Q) heated to 363 K. Ammonium hydroxide (28%, VWR) was added dropwise very slowly with rigorous stirring until the solution reached a pH of 10, generating a fatty acid soap. Magnesium stearate was precipitated by adding 0.1 mol of MgCl_2_·6H_2_O (99%, Sigma–Aldrich). Finally, the product was isolated by vacuum filtration and suspended for 24 h in acetone and then for 24 h in de­ionized water. Vacuum filtration and drying of the suspended powder, resulted in a fluffy white powder. The yield was not precisely measured as it was not expected to influence the findings.

### 2.2. Scanning electron microscopy

Scanning electron microscopy (SEM) was performed using a QUANTA FEG 3D (Thermo Fischer Scientific, USA). The microscope was used in high-vacuum mode with an electron beam with a diameter of 1.2 nm at 30 kV with an Everhart–Thornley detector.

### 2.3. X-ray diffraction data collection of the micrometre-sized crystals

**Figure 2:**
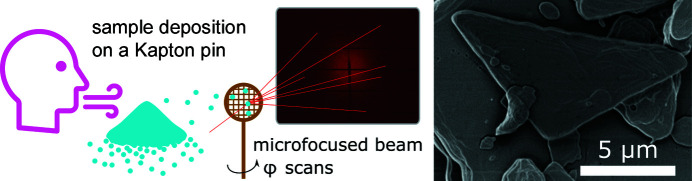
(Left) The sample preparation and diffraction experiment strategy. (Right) An SEM image of magnesium stearate single crystals.

SEM images of the purified magnesium stearate powder showed micrometre-sized plate-like crystals tentatively suited to a diffraction experiment (see representative image in Fig. 2 ▸). The crystals were blown onto dedicated Kapton pins (MiTeGen) and inspected under an optical microscope to find the best candidates, *i.e.* pins with a few deposited individual crystals rather than denser powder clumps. Selected samples were immersed in liquid nitrogen for transport to the MAX IV synchrotron facility (BioMAX beamline) in Lund, Sweden (Ursby *et al.*, 2020 ▸). In fact, the beamline robot handling the samples and data collection was fully controlled online from the University of Copenhagen in Denmark due to the global Covid-19 pandemic.

For the diffraction data collection, a microfocused beam (5 × 20 µm FWHM threaded through a 10 µm circular aperture, λ = 0.787201 Å) was used to collect φ scans. The software *XDS* (Kabsch, 2010 ▸) was used for data indexing. Out of over forty diffraction data collections only one could be successfully indexed. Subsequent integration and reduction of the data was performed using *XDS*. The Laue group 2/*m* was applied for averaging of the reflections.

### 2.4. Structure solution and refinement

The software *JANA2006* (Petříček *et al.*, 2014 ▸) was used for structure solution and refinement. Anisotropic displacement parameters were refined for non-hydrogen atoms. The hydrogen atoms of the stearate alkyl chain were fixed at calculated positions and refined using a riding model, where the H atom positions are constrained to the idealized geometry of the nearest three carbon atoms. Due to the weak diffraction of the analysed single crystal, the data resolution was not high {[sin(θ)/λ]_max_ = 0.571 Å^−1^} and the completeness in the last resolution shell (0.52 to 0.57 Å^−1^) was only 85%. Only 28% of all reflections were classified as observed.

A range of models were tried in order to position and refine the hydrogen atoms of the water molecules. Five different models were tested, where the water molecule was defined as a rigid body with a fixed H—O—H angle of 104.4° and allowing the O—H distance to be refined, but keeping the O—H distances equal within 0.005 s.u. The refinements were performed with different starting orientations of the three water molecules. The models all converge and provide very similar refinement indices. All details of these models can be found in the supporting information. The conclusion is that the hydrogen atoms of the water molecules could not be located, nor could their positions be reliably refined.

Since it is not possible to fix the positions of those hydrogen atoms due to the unknown orientation of the water molecules, the refinement was performed without the hydrogen atoms, but the occupancy of the water oxygen atoms was set to 1.25 to take into account the electron density of the two missing hydrogen atoms, which we believe is commensurate with the quality of the available data set. Complete refinement details are listed in Table 1 ▸.

The refined structure and data have been deposited with the Cambridge Crystallographic Data Centre (deposition number 2125037) and in the Crystallography Open Database (deposition number 3000423).

### 2.5. Periodic dispersion-corrected DFT calculations

As described above, the limited data resolution did not allow us to locate the hydrogen atoms of the water molecules. A geometry optimization of the structure using periodic dispersion-corrected density functional theory (DFT) calculations was performed in order to obtain the positions of the missing hydrogen atoms. This resulted in a plausible hydrogen-bonding network throughout the structure.

Hydrogen atoms were added to the water oxygen atoms with plausible positions and then the geometry was optimized using the COMPASS force field (Sun, 1998 ▸), while fixing all the other atomic positions and the unit-cell parameters. Placement of the hydrogen atoms at different starting positions resulted in the same hydrogen-bond pattern after the force-field optimization. The resulting structure model was further optimized using DFT with the *VASP* code (Kresse & Hafner, 1993 ▸), including the optimization of the unit-cell parameters. The Perdew–Burke–Ernzerhof density functional (Perdew *et al.*, 1996 ▸) was used with the Grimme D3 dispersion correction (Grimme *et al.*, 2010 ▸) which is crucial for correctly addressing the energy of molecular crystalline systems (van de Streek & Neumann, 2010 ▸). The structures were optimized with convergence criteria of 5 × 10^−6^ eV for the electronic relaxation and 5 × 10^−5^ eV for the ionic relaxation, and with a plane-wave basis set cutoff energy of 520 eV. A 2 × 2 × 2 *k*-point grid was used. The CIF containing this optimized structure model with all hydrogen atoms is available in the supporting information.

## 3. Results and discussion

**Figure 3:**
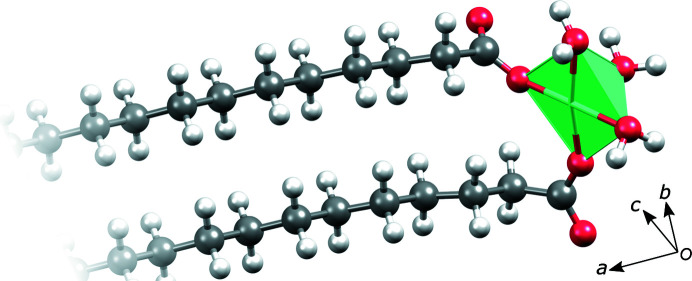
The asymmetric unit of the magnesium stearate trihydrate structure, focused on the metal coordination.

The asymmetric unit of magnesium stearate trihydrate contains one magnesium ion which is octahedrally coordinated by three water molecules and two stearate anions through the carboxyl oxygen atoms (Fig. 3 ▸). This indicates that the particular structure corresponds to a trihydrate, Mg(C_17_H_35_COO)_2_·3H_2_O. The sixth coordination site is occupied by another stearate oxygen atom, related by space-group symmetry. A magnesium ion meridionally coordinated by three water molecules and three carboxyl oxygen atoms, as found in this particular structure, seems to be the thermodynamically most favourable state in systems with a low dielectric constant (Dudev *et al.*, 1999 ▸). It is preferred over octahedrally coordinated states with different numbers of water molecules and carboxylic acid groups. Bidentate binding of carboxylate to magnesium also seems to be slightly unfavourable when replacing a water molecule (Dudev & Lim, 2004 ▸).

**Figure 4:**
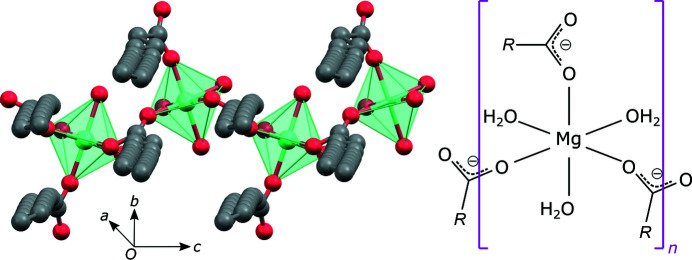
(Left) Polymeric chains of the coordinated magnesium octahedra resulting from the expansion of the donor–acceptor bond network. (Right) A schematic representation of the corresponding structural unit (*R* = C_17_H_35_).

In the crystal structure, further expansion of the donor–acceptor bond network results in a slab in which there is an infinite polymeric chain of coordinated magnesium octahedra (left-hand side of Fig. 4 ▸). The octahedra do not share faces, edges or vertices. They are linked through stearate carboxyl bridges, as clearly indicated in the right-hand side of Fig. 4 ▸.

**Figure 5:**
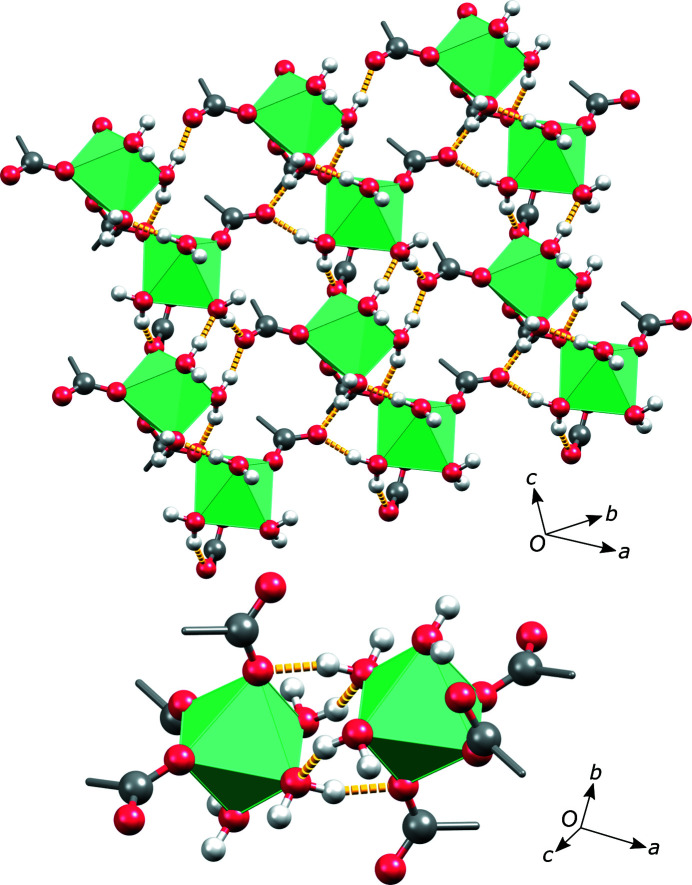
(Top) The polar side of the layer in the magnesium stearate trihydrate structure, showing the hydrogen bonding. (Bottom) The hydrogen bonding connecting two polar faces of the layers. Stearate alkyl chains have been omitted for clarity.

**Figure 6:**
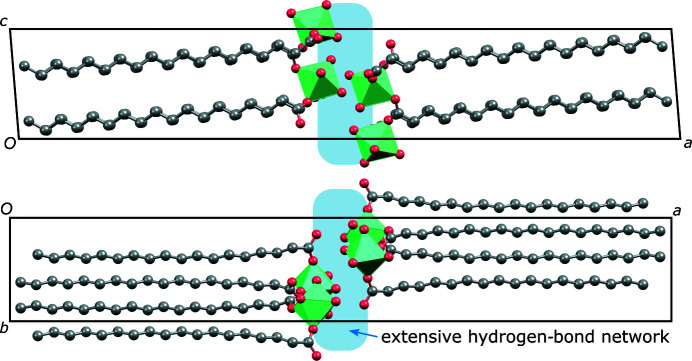
Overall crystal structure representations of magnesium stearate tri­hydrate as viewed along (top) the *b* axis and (bottom) the *c* axis.

Our DFT-optimized model allows us to explore the hydrogen bonding in this structure. Hydrogen bonding is present between the water molecules on the octahedra vertices and non-coordinated oxygen atoms of the stearate anions. Some of these bonds interconnect the donor–acceptor-bonded slabs to form an infinite layer with a polar and a non-polar side (top of Fig. 5 ▸). Each pair of layers in the crystal structure interact face-to-face through their polar sides and there are additional hydrogen bonds connecting these faces (bottom of Fig. 5 ▸). The overall packing of the magnesium stearate trihydrate is depicted in Fig. 6 ▸.

The structure has some similarities with previously published structures of other metal soaps, *e.g.* calcium stearate (Lelann & Bérar, 1993 ▸), potassium palmitate (Dumbleton & Lomer, 1965 ▸) and silver behenate (Blanton *et al.*, 2011 ▸). In all those structures, the metal ions are coordinated by the carboxyl oxygen atoms and form carboxylate double layers with the aliphatic chains facing outwards. Therefore, the remaining magnesium stearate anhydrous and hydrated phases for which the exact structures are not yet known are expected to be similarly organized.

We allow ourselves to speculate about the mechanism behind the change in lubricating properties of magnesium stearate as a function of hydration level. Lubrication is a key functionality of magnesium stearate in the context of tablett­ing pharmaceuticals. There is a strong thermodynamic incentive for monodentate coordination of three carboxylic groups and three water molecules to the magnesium ion (Dudev *et al.*, 1999 ▸). As described previously, this is the case in the trihydrate phase (right-hand side of Fig. 4 ▸). In the di-, mono- and anhydrates, the number of water molecules that can act as ligands is insufficient to maintain a coordination number of six, unless the water molecules act as bridging ligands for two magnesium ions, or unless some of the carboxylates act as bidentate ligands. However, several bidentate carboxylate ligands will sterically hinder the packing seen in the trihydrate. The more likely role of bridging water molecules will lead to vertice- or edge-sharing coordination polyhedra in the structure, and may preserve the overall packing motif, albeit with a probable stronger binding across the polar interface, thus changing the shearing properties of the crystals and thereby leading to changing lubrication properties.

The determined structure of magnesium stearate trihydrate will serve as a source for an in-depth mechanistic understanding of the lubricating properties of this extremely widely used pharmaceutical additive, *e.g.* with computational techniques such as molecular dynamics. We also believe that the obtained structure can be used as a starting point for construction of plausible structural models of the other hydrate and anhydrate phases of magnesium stearate. As noted above, the magnesium–carboxylate bonds are very strong, and this constrains the possible architectures of the polar region of the crystals. At the same time, other structures of metal soaps indicate that the aliphatic chains and the non-polar parts of the structures are structurally similar. It is very likely that a combination of models based on the determined trihydrate and high-resolution powder diffraction can provide the structures of the remaining hydrates.

## 4. Conclusion

This study shows that classical single-crystal X-ray diffraction performed at a high-brilliance synchrotron facility is suitable for crystal structure determination, even if the samples are considerably smaller than conventionally deemed to be satisfactory for such experiments. In addition, DFT calculations can be used to enhance further the structure determined from lower-resolution data than usually desired.

The determined structure of magnesium stearate trihydrate will serve as a source for improving our understanding of the lubricating properties of this extremely widely used pharmaceutical additive, and it can aid in the determination of other similar structures (lower hydrates of magnesium stearate or magnesium palmitate hydrates) from *e.g.* powder X-ray diffraction data.

## Supplementary Material

Crystal structure: contains datablock(s) MgSt_3H2O. DOI: 10.1107/S2052520623005607/rm5068sup1.cif

Structure factors: contains datablock(s) MgSt_3H2O. DOI: 10.1107/S2052520623005607/rm5068MgSt_3H2Osup2.hkl

Bilbao data. DOI: 10.1107/S2052520623005607/rm5068sup3.txt

Details of the five hydrogen-bonding models. DOI: 10.1107/S2052520623005607/rm5068sup4.pdf

ZIP archive of CIFs and structure factors for hydrogen-bonding models. DOI: 10.1107/S2052520623005607/rm5068sup5.zip

CCDC reference: 2125037

## Figures and Tables

**Table 1:** Relevant crystallographic data

*T* (K)	100
Formula	Mg(C_17_H_35_COO)_2_·3H_2_O
Formula weight (g mol^−1^)	645.3
Crystal system	Monoclinic
Space group	*P*2_1_/*c*
*a*, *b*, *c* (Å)	53.1859 (9), 8.219 (17), 8.891 (6)
β (°)	94.690 (8)
*V* (Å^3^)	3873.5 (2)
*Z*, *Z*′	4, 1
*F*(000)	1440
*D*_*x*_ (g cm^−3^)	1.1065
μ (mm^−1^)	0.108
	
No. of measured reflections, unique and observed reflections [*I* > 3σ(*I*)]	17686, 5127, 1443
(sin θ)/λ)_max_ (Å^−1^)	0.571
*R*_int_ (observed, all)	0.1074, 0.2128
	
Refinement method	Full-matrix least-squares on *F*
No. of parameters	397
*R*_1_ (observed, all)	0.0777, 0.2577
*wR* (observed, all)	0.0891, 0.1118
GoF (observed, all)	2.19, 1.31
H-atom treatment	See text
Weighting scheme	*w* = 1/[σ^2^(*F*) + 0.0001*F* ^2^]
Δρ_max_, Δρ_min_ (e Å^−3^)	0.37, −0.42
