# Supplementary material for: The structure of magnesium stearate trihydrate determined from a micrometre-sized single crystal using a microfocused synchrotron X-ray beam
Source: Acta Crystallogr B Struct Sci Cryst Eng Mater. 2023 Jul 8;79(Pt 4):330–5. doi: 10.1107/S2052520623005607 (PMC10410307; doi:10.1107/S2052520623005607)
Supplement: Supplementary file 4 [file b-79-00330-sup4.pdf]

STRUCTURAL SCIENCE
CRYSTAL ENGINEERING
MATERIALS

Volume 79 (2023)

Supporting information for article:

The structure of magnesium stearate trihydrate determined from a micrometre-sized single crystal using a microfocused synchrotron X-ray beam

Mikkel Herzberg, Toms Rekis, Anders Støttrup Larsen, Ana Gonzalez, Jukka Rantanen and Anders Østergaard Madsen

Supporting Information

The structure of magnesium stearate, determined from a micron-size single-crystal using synchrotron beam

Mikkel Herzberg[1]+, Toms Rekis[1]+, Anders S. Larsen[1], Ana Gonzalez[2], Jukka Rantanen[1] and Anders Ø. Madsen[1]*

[1] MSc Mikkel Herzberg, Dr. Toms Rekis, Dr. Jukka Rantanen and Dr. Anders Ø. Madsen*
Department of Pharmacy
University of Copenhagen
Universitetsparken 2, 2100 Copenhagen, Denmark
E-mail: a.madsen@sund.ku.dk

[2] Dr. Ana Gonzalez
BioMAX
MAX IV
Fotongatan 2, 224 84 Lund, Sweden

[+] These authors contributed equally to the work (co-first authors)

Hydrogen atoms for water molecules: five different models

Probed by an interesting discussion with an anonymous referee, we have tested whether hydrogen atoms for the water molecules could be reliably refined. We have defined the water molecules as rigid bodies with fixed H-O-H angles of 104.4 deg and allowing the O-H distances to be refined, but keeping both O-H distances equal within 0.005 s.u. For each water molecule, 6 parameters are used to refine the position and orientation of the *quasi*-rigid body fragment. We have done 5 refinements in program Jana2006 with different starting orientations of the 3 water molecules. In the picture below, all structures are overlaid (including the one offered by the referee, refined in Shelxl, in magenta). As it can be seen, the hydrogen positions can be very different.

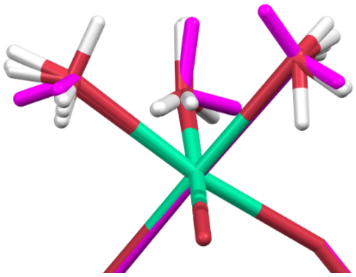

All refinements have converged without errors. Low R values are obtained for all refinements (see the table below). We have used I > 2sigma for these refinements.

	Referee	#1	#2	#3	#4	#5
Reflections (obs.)	1778	1761	1761	1761	1761	1761
Parameters	403	397	397	397	397	397
R1 (obs.)	0.1157	0.0876	0.0870	0.0869	0.0870	0.0874
R1 (all)	0.2560	0.2515	0.2515	0.2523	0.2518	0.2531
wR (obs.)	0.2646	0.0917	0.0908	0.0902	0.0907	0.0914
wR (all)	0.3746	0.1087	0.1080	0.1073	0.1080	0.1087
GoF	0.951	1.27	1.26	1.25	1.26	1.27

It is clear that the positions of the hydrogen atoms cannot be determined reliably in this case since several options with very different positions give nearly identical R-values. Jana2006 refinement files as well as CIF and FCF files are supplied as supporting information.
